# Evaluating Cell-Free DNA Quantification and Integrity in Breast Cancer Using Fluorometric, Electrophoretic and PCR-Based Platforms

**DOI:** 10.3390/mps9050124

**Published:** 2026-08-24

**Authors:** Gisha Rose Antony, Vidya P. Nimbalkar, Sowmya Nagaraj, Mahalakshmi Subramanya, Anupama C. Eshwara Rao, Ganapathi Bantwal, Belinda George, Akshay Ajoy, Nihal M. Ahamed, Rakesh S. Ramesh, Jyothi D. N, Jaya Kumari S, Jyothi S. Prabhu

**Affiliations:** 1 Division of Molecular Medicine, St. John’s Research Institute (SJRI), Bangalore 560034, India; rosekakkanattu@gmail.com (G.R.A.); sowmya.sn@sjri.res.in (S.N.);; 2 Department of Endocrinology, St John’s National Academy of Health Sciences (SJMCH), Bangalore 560034, Indiabelinda.g@stjohns.in (B.G.); 3 St. John’s Medical College (SJMC), Bangalore 560034, Indianihalmalayil@gmail.com (N.M.A.); 4 Department of Surgical Oncology, St John’s National Academy of Health Sciences (SJMCH), Bangalore 560034, India; 5 Department of Biochemistry, St. John’s Medical College (SJMC), Bangalore 560034, Indiajayakumari.s@stjohns.in (J.K.S.)

**Keywords:** breast cancer, cell-free DNA, cfDNA integrity, liquid biopsy

## Abstract

Cell-free DNA (cfDNA) has emerged as a promising liquid biopsy biomarker; however, differences in analytical methods may influence cfDNA quantification and integrity assessment. This study compared plasma cfDNA concentration and integrity between treatment-naïve breast cancer (BC) patients (*n* = 100) and healthy controls (HC; *n* = 70) using complementary analytical platforms. cfDNA concentration was measured by Qubit fluorometry, TapeStation (TS) electrophoretic profiling, and ALU-based quantitative PCR (qPCR). An exploratory genomic DNA (gDNA) correction of Qubit-derived cfDNA concentration was performed using TS-derived fragment-size information. cfDNA integrity was assessed using the ALU-derived DNA Integrity Index (DII (ALU); ALU247/ALU115) and the TS-derived DNA Integrity Index (DII (TS)), and methods were compared for their correlation and agreement. Qubit- and TS-based cfDNA measurements were significantly higher in BC patients than in HC. Among the ALU-based qPCR measures, although ALU115 did not differ significantly between groups, ALU247 and DII (ALU) were significantly higher in BC patients, indicating an enrichment of longer cfDNA fragments. Exploratory gDNA correction did not substantially alter the observed differences. Significant positive correlations were observed among the cfDNA quantification methods. Supporting these findings, agreement analysis demonstrated improved agreement following gDNA correction. In contrast, cfDNA integrity assessment was method-dependent: although DII (ALU) was significantly higher in BC patients, DII (TS) did not differ significantly between groups, and no significant correlation or agreement was observed between the two indices, indicating that the two methods evaluate different lengths of cfDNA fragments. These findings demonstrate that complementary analytical platforms consistently differentiated BC patients from HC based on cfDNA concentration, whereas cfDNA integrity assessment depended on the analytical method employed. Together, these findings provide methodological insights into cfDNA quantification and fragmentation assessment and support the complementary use of fluorometric, electrophoretic, and ALU-based qPCR approaches for comprehensive cfDNA characterization.

## 1. Introduction

Cell-free DNA (cfDNA) refers to short DNA fragments released into the bloodstream through apoptosis, necrosis, or active cellular secretion. In patients with malignancy, a fraction of this circulating DNA originates from tumor cells (circulating tumor DNA, ctDNA), reflecting tumor dynamics, burden, and genomic alterations [1,2,3]. ctDNA is identified within cfDNA by tumor-specific mutations, copy-number variations, gene rearrangements, and methylation signatures [4]. Its ability to detect cfDNA through a simple blood draw has made it a central analyte in liquid biopsy-based cancer diagnostics [5]. In breast cancer (BC), elevated cfDNA levels and altered fragmentation profiles have been associated with advanced stage, poor prognosis, and minimal residual disease [6,7,8], highlighting cfDNA as a potential non-invasive biomarker both for early detection and disease monitoring.

Both the quantitative and qualitative characteristics of cfDNA are informative. The total cfDNA concentration reflects global DNA release and tumor cell turnover [9,10], whereas the cfDNA integrity index (DII), defined as the ratio of long to short DNA fragments, captures the underlying mode of cell death [6,7]. High DII values have been linked to an increased abundance of longer cfDNA fragments that may reflect tumor-associated cfDNA features, whereas low DII values are generally associated with the shorter apoptotic fragmentation patterns observed in healthy individuals [7,11,12]. Reliable measurement of cfDNA concentration and integrity is therefore essential for distinguishing tumor-derived cfDNA from background DNA of hematopoietic origin.

However, cfDNA analysis is complicated by methodological diversity. Fluorometric assays such as Qubit quantify total double-stranded DNA rapidly but cannot discriminate cfDNA from contaminating gDNA. Electrophoretic instruments like the Agilent TS visualize cfDNA fragment size distribution, identifying mono- and di/tri-nucleosomal fragments, yet they may fail to detect highly degraded cfDNA [13,14]. In contrast, qPCR assays targeting repetitive elements such as ALU sequences enable sequence-specific quantification and DII calculation (e.g., ALU 247/ALU 115), offering higher analytical sensitivity [15]. Reported discrepancies among these approaches raise concerns about assay comparability and interpretation of cfDNA-based biomarkers.

Only a limited number of studies have directly compared cfDNA quantification and integrity across multiple analytical platforms, and virtually none have done so in a treatment-naïve Indian BC cohort. To address this gap, we performed a systematic comparison of cfDNA concentration and integrity in plasma from BC patients and healthy controls using three complementary techniques, Qubit fluorometry, TS electrophoretic profiling, and ALU-based qPCR. We further incorporated gDNA-correction of fluorometric data and assessed inter-method concordance. This work aims to identify the most reliable and sensitive approach for cfDNA assessment and to provide practical guidance for assay standardization in BC liquid-biopsy research.

## 2. Materials and Methods

### 2.1. Patients and Healthy Control Samples

Peripheral blood samples were obtained from 100 female patients with histologically confirmed primary breast cancer. Samples were collected prior to the initiation of any cancer-directed treatment, after obtaining written informed consent. Inclusion criteria for the patient cohort were histologically confirmed primary breast cancer, age ≥ 18 years, availability of a pre-treatment blood sample, and no prior cancer-directed therapy. Healthy volunteers (*n* = 70) aged ≥ 18 years, including both males and females, with no history of malignancy or known comorbidities, were recruited as healthy controls (HC). The study was approved by the Institutional Ethics Committee, and written informed consent was obtained from all participants.

Peripheral blood samples were collected in EDTA tubes and processed within 2 h of collection. Plasma was separated by centrifugation at 1300× *g* for 10 min at 4 °C and carefully aliquoted to minimize cellular contamination. Plasma aliquots were stored at −80 °C in the institutional biorepository until cfDNA extraction. Repeated freeze–thaw cycles were avoided to minimize cfDNA degradation and preserve fragment integrity.

### 2.2. cfDNA Extraction

cfDNA from BC patients and HC samples was extracted from 500 µL of plasma using the cfPure^®^ Cell-Free DNA Extraction Kit (#K5011610, BioChain Institute, Inc., Newark, CA, USA) according to the manufacturer’s instructions. The extracted cfDNA was eluted in 30 µL of elution buffer and was quantified using the Qubit™ dsDNA High Sensitivity (HS) DNA Assay Kit (#Q32851, Thermo Fisher Scientific, Waltham, MA, USA) on a Qubit™ 4 Fluorometer (Thermo Fisher Scientific) and stored at −20 °C. cfDNA concentrations were measured in the 30 µL elute and are reported throughout the manuscript as ng/µL. For comparison with studies expressing cfDNA concentration as ng/mL of plasma, the following conversion can be applied:

$$\mathrm{cfDNA}\;\mathrm{concentration}\;\left(\mathrm{ng}/\mathrm{mL}\right)\;=\;\frac{\mathrm{Qubit}\;\mathrm{concentration}\;\left(\mathrm{ng}/\mu L\right)\;\times \;\mathrm{Elution}\;\mathrm{volume}\;\left(30\;\mu L\right)\;\times \;1000}{\mathrm{Input}\;\mathrm{plasma}\;\mathrm{volume}\;\left(500\;\mu L\right)}$$

This simplifies to:

cfDNA concentration (ng/mL) = Qubit concentration (ng/µL) × 60

### 2.3. cfDNA Fragmentation Profiling and DII Calculation

The fragmentation profile of cfDNA was analyzed using the TS4150 system (Agilent Technologies, Santa Clara, CA, USA). For each analysis, 2 μL of extracted cfDNA was mixed with 2 μL of HS D1000 sample buffer (#5067-5603, Agilent Technologies) in an 8-tube strip. The HS D1000 Ladder (#5067-5587, Agilent Technologies), prepared according to the manufacturer’s instructions, was loaded into well A1 of the tube strip holder for fragment size calibration.

Electropherograms were analyzed using Agilent TSAnalysis Software version 5.1 (Agilent Technologies). Fragment size regions were predefined as 101–300 bp, representing predominantly mono-nucleosomal (short) cfDNA fragments, and 301–1000 bp, representing predominantly di- and tri-nucleosomal (long) cfDNA fragments. DNA concentrations within each fragment-size region were automatically quantified by the TapeStation Analysis Software version 5.1 (Agilent Technologies) based on the integrated electropherogram peak area (area under the curve) following calibration with the HS D1000 ladder. Concentrations were calculated using automated peak detection, baseline correction, background subtraction, and signal integration. No manual adjustment of fragment-size boundaries or additional baseline correction was performed.

The TS-derived DNA Integrity Index (DII (TS)) was calculated as the ratio of the DNA concentration within the long-fragment region (301–1000 bp) to that within the short-fragment region (101–300 bp):

$$\mathrm{DII}\;\left(\mathrm{TS}\right)\;=\frac{\mathrm{DNA}\;\mathrm{concentration}\;\left(301-1000\;\mathrm{bp}\right)}{\mathrm{DNA}\;\mathrm{concentration}\;\left(101-300\;\mathrm{bp}\right)}$$

### 2.4. gDNA Correction of the cfDNA Total Concentration

To obtain an exploratory estimate of cfDNA concentration after accounting for residual genomic DNA (gDNA) contamination, the Qubit-derived total DNA concentration was adjusted using fragment-size information obtained from the TS electropherogram. The TS electropherogram provides the distribution of DNA fragment sizes, enabling estimation of the proportion of high-molecular-weight DNA within each sample. The percentage of DNA fragments >1 kb was used as an operational estimate of the high-molecular-weight DNA fraction, which may predominantly represent residual genomic DNA contamination. This estimated fraction was subsequently used to adjust the total DNA concentration measured by the Qubit fluorometer [16] according to the following formula:

Corrected cfDNA concentration = Qubit total DNA concentration × (1 − gDNA%)

where the gDNA% corresponds to the proportion of DNA fragments >1 kb measured from the TapeStation electropherogram.

### 2.5. Estimation of cfDNA Concentration and DII by qPCR

Absolute quantification of cfDNA was performed by quantitative PCR (qPCR) targeting ALU repetitive elements, as previously described [15]. Briefly, 1 µL of template DNA was added to a 10 µL reaction volume containing 3 pmol of each primer. Amplification was performed using SYBR Green master mix (Takara Bio Inc., Kusatsu, Shiga, Japan) on the LightCycler ^®^ 480 II platform (Roche Diagnostics GmbH, Mannheim, Germany) using the optimized assay conditions described previously [15]. Absolute quantification was achieved using a standard curve generated from serial dilutions (10 ng to 0.01 pg) of commercially available human genomic DNA (#G3041, Promega Corporation, Madison, WI, USA). ALU115 and ALU247 concentrations were calculated by interpolation from the corresponding standard curves. A no-template control (NTC) was included in each qPCR run to monitor contamination. Detailed qPCR assay conditions, quality control measures, and assay validation information are provided in the Supplementary Methods.

The primer pair used is as follows:

ALU 115 Forward: 5′-CCTGAGGTCAGGAGTTCGAG-3′

Reverse: 5′-CCCGAGTAGCTGGGATTACA-3′

ALU 247 Forward: 5′-GTGGCTCACGCCTGTAATC-3′

Reverse: 5′-CAGGCTGGAGTGCAGTGG-3′

The ALU-derived DNA integrity index (DII (ALU)) was calculated as the ratio of ALU247 to ALU115 concentrations (ALU247/ALU115). Both ALU targets were quantified independently by qPCR using standard curves, as previously described [15].

### 2.6. Assessment of Residual Genomic DNA Influence on ALU-Based Measurements

To further evaluate the potential influence of residual genomic DNA contamination on ALU-based biomarkers, the estimated genomic DNA fraction (gDNA%) for each sample was calculated from the Qubit-derived total DNA concentration and gDNA-corrected cfDNA concentrations using the formula:

$$\mathrm{gDNA}\%\;=\;\frac{\text{Qubit-derived}\;\mathrm{total}\;\mathrm{DNA}\;\mathrm{concentration}-\mathrm{Corrected}\;\mathrm{cfDNA}\;\mathrm{concentration}}{\mathrm{Qubit}\;\mathrm{concentration}}\;\times \;100$$

Spearman correlation analysis was performed to evaluate the association between the estimated gDNA% and ALU247 concentration as well as the DII (ALU). In addition, group comparisons of ALU247 concentration and the DII (ALU) between BC patients and (HC) were performed using the complete dataset and repeated after exclusion of the 5% and 10% of samples with the highest estimated genomic gDNA% to assess the robustness of the findings. In addition, exploratory linear regression analyses were performed separately for ALU247 and DII (ALU), with sample group (BC vs. HC) and estimated gDNA% included as covariates, to determine whether the group differences persisted after adjustment for estimated gDNA%.

### 2.7. Statistical Analysis

Statistical analyses were performed using R (version 4.5.1). Differences in cfDNA concentration and DNA integrity index (DII) between HC and BC patients were assessed using the Mann–Whitney U test, and effect sizes were reported as Hodges–Lehmann median differences with corresponding 95% confidence intervals (CI) and rank-biserial correlation coefficients. Effect size estimates are provided in the respective tables, where appropriate. Exploratory linear regression analyses were performed separately for ALU247 and DII (ALU), with sample group (Tumor vs. Control) and estimated gDNA% included as covariates.

Associations between cfDNA quantification methods, as well as between the ALU-derived and TS-derived DNA integrity indices, were evaluated using Spearman’s rank correlation coefficient. Further, Bland–Altman analysis was additionally performed to evaluate agreement between the different cfDNA quantification methods and between the ALU-derived and TS-derived DNA integrity indices by estimating the mean bias and 95% limits of agreement (LoA). Comparisons involving more than two groups were performed using the Kruskal–Wallis test. For the prespecified subgroup analyses (age, tumor stage, and molecular subtype), the Benjamini–Hochberg (BH) procedure was applied to the overall *p* values to control the false discovery rate. A two-sided *p* < 0.05 was considered statistically significant unless otherwise specified.

## 3. Results

### 3.1. cfDNA Concentration Is Elevated in BC Patients Compared to HC

**Figure 1:**
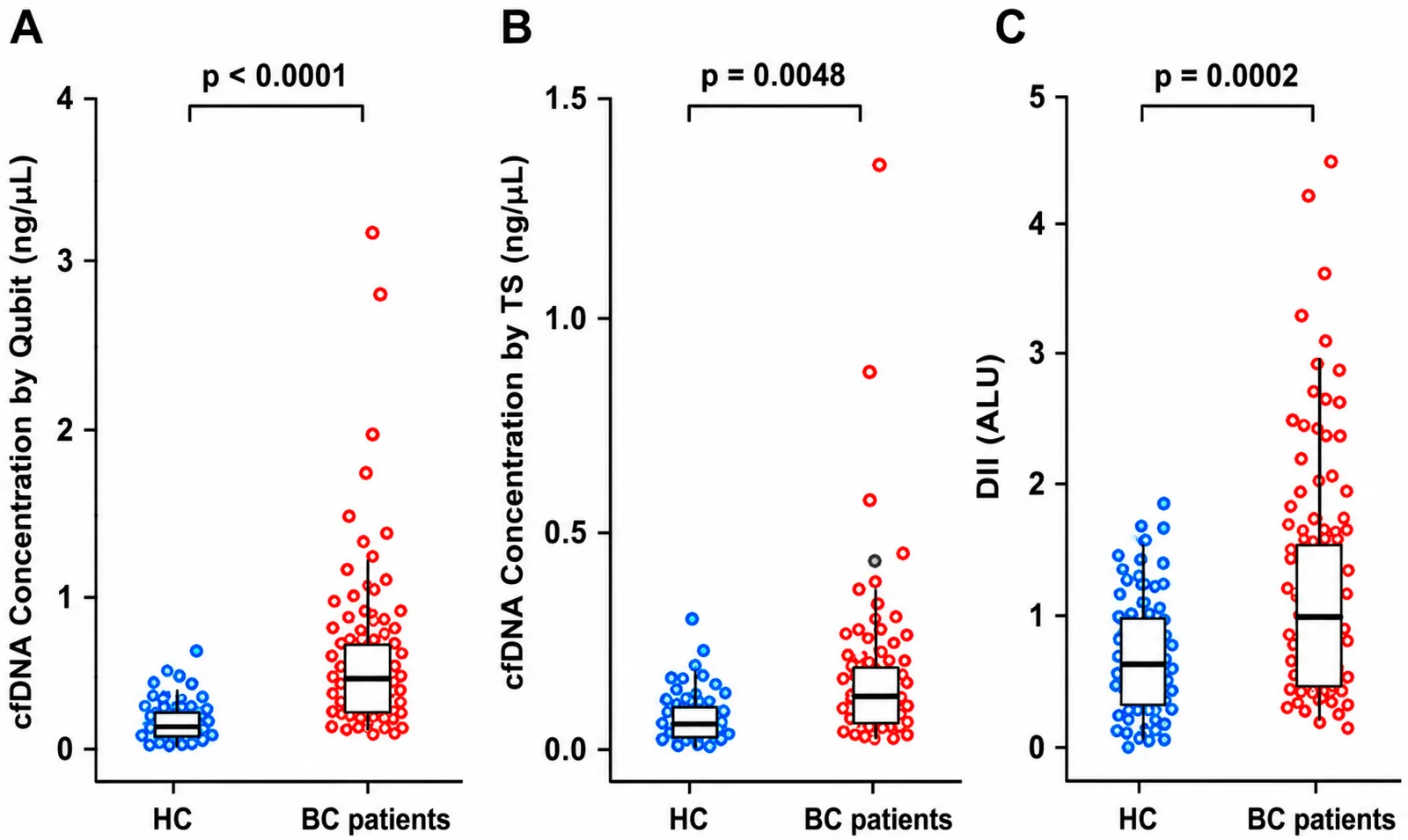
Comparison of cfDNA levels and DII between BC patients and HC. (**A**) Total cfDNA concentration measured by Qubit fluorometry shows a significant increase in BC patient samples compared to HC (*p* < 0.0001). (**B**) cfDNA concentration estimated by TS electrophoretic profiling also demonstrates higher levels in BC patients (*p* = 0.0048). (**C**) ALU-derived DNA Integrity Index [DII (ALU)] is significantly higher in BC patients, indicating a greater relative abundance of longer amplifiable cfDNA fragments (*p* = 0.0002). Individual observations are shown as dots; the box represents the interquartile range (IQR; Q1–Q3), and the horizontal line indicates the median. *p*-values were obtained using the Mann–Whitney U test.

A total of 170 plasma samples (100 BC patients and 70 healthy controls [HC]) were included in the analyses (Supplementary Table S1). cfDNA concentration was first assessed using Qubit fluorometry and TS electrophoretic profiling. Qubit-derived cfDNA concentrations were significantly higher in BC patients than in HC (median [Q1–Q3]: 0.329 [0.245–0.490] vs. 0.240 [0.174–0.300] ng/µL, *p* < 0.0001) (Figure 1A, Supplementary Table S2). Similarly, TS-derived cfDNA concentrations were significantly higher in BC patients than in HC (median [Q1–Q3]: 0.109 [0.065–0.170] vs. 0.084 [0.053–0.110] ng/µL, *p* = 0.0048) (Figure 1B, Supplementary Table S2).

cfDNA concentrations were further explored across clinically relevant subgroups, including age (>50 vs. ≤50 years), tumor stage (early vs. late), and molecular subtype (HER2+, HR+, and TNBC). Median cfDNA concentrations varied numerically among the molecular subtypes, with the highest values observed in HR+ tumors (median [Q1–Q3]: 0.351 [0.247–0.501] ng/µL), followed by HER2-positive tumors (0.330 [0.252–0.450] ng/µL) and TNBC (0.256 [0.201–0.351] ng/µL). However, the overall Kruskal–Wallis test did not identify significant differences among the molecular subtypes. Likewise, no statistically significant differences in cfDNA concentrations were observed across age groups, tumor stage, or molecular subtypes after Benjamini–Hochberg (BH) adjustment for multiple comparisons (adjusted *p* = 0.100 for all comparisons). Consistent with these findings, effect size estimates were small across all subgroup analyses (Supplementary Figure S1 and Supplementary Table S3).

**Figure 2:**
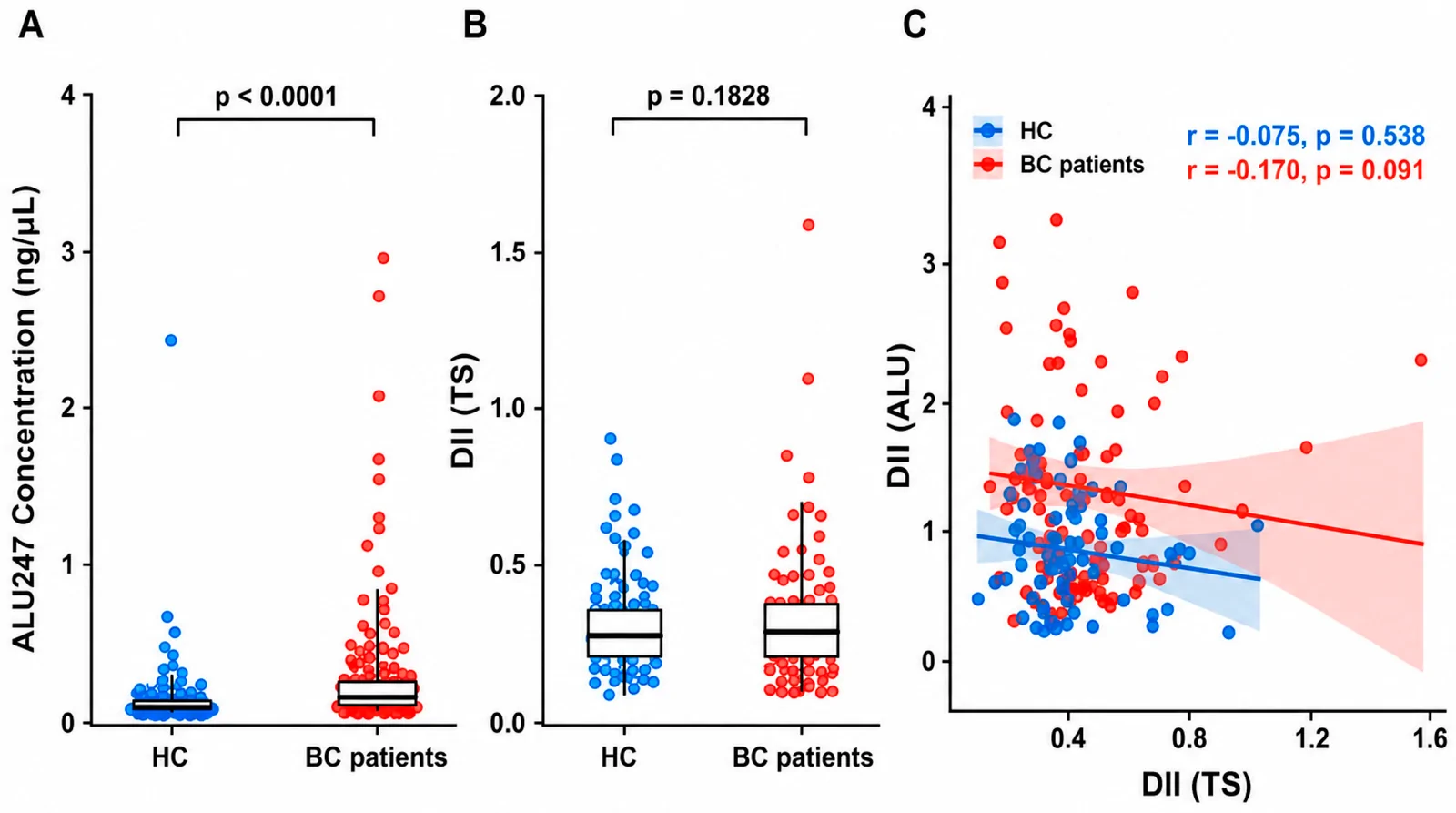
Comparison of ALU247 concentration and cfDNA integrity indices between HC and BC patients. (**A**) Distribution of ALU247 concentration in HC and BC patients. ALU247 concentration was significantly higher in BC patients than in HC (*p* < 0.0001). (**B**) Distribution of TS-derived DNA integrity index [DII (TS)] in HC and BC patients. No significant difference was observed between the groups (*p* = 0.1828). (**C**) Correlation between ALU and TS-derived integrity indices [DII (ALU) and DII (TS)] in HC and BC patients. No significant association was observed between the two methods (Spearman’s rank correlation: HC, r = −0.075, *p* = 0.54; BC patients, r = −0.170, *p* = 0.091). For boxplot panels (**A**,**B**), individual observations are shown as dots; the box represents the interquartile range (IQR; Q1–Q3), and the horizontal line indicates the median. *p*-values were obtained using the Mann–Whitney U test. HC: Healthy Controls, BC: Breast Cancer; TS: TapeStation.

ALU repetitive elements were subsequently quantified by qPCR. ALU247 concentrations were significantly higher in BC patients than in HC (median [Q1–Q3]: 0.113 [0.070–0.228] ng/µL vs. 0.055 [0.039–0.079] ng/µL; *p* <0.0001; Figure 2A, Supplementary Table S2), whereas ALU115 concentrations did not differ significantly between the two groups (*p* = 0.218; Supplementary Figure S2). Consistent with these findings, the ALU-derived DNA Integrity Index (DII (ALU); ALU247/ALU115) was significantly higher in BC patients than in HC (median [Q1–Q3]: 1.074 [0.531–1.522] vs. 0.714 [0.364–1.040]; *p* = 0.0002; Figure 1C, Supplementary Table S2).

### 3.2. TS Profiles Showed Visible gDNA Contamination in Both Groups

We next evaluated cfDNA fragment distributions in the BC patients and HC groups using TS electrophoretic profiling. The fragment analysis revealed detectable gDNA contamination in both BC patients and HC samples (Supplementary Figure S3A–F). To assess the extent to which gDNA contamination contributed to the measured cfDNA concentrations, we performed gDNA correction on the Qubit-estimated concentrations. After subtracting the gDNA% determined from the TS profiles, the median corrected cfDNA concentrations remained significantly higher in BC patients than in HC (median [Q1–Q3]: 0.195 (0.132–0.284) ng/µL vs. 0.150 (0.093–0.191) ng/µL, *p* < 0.0001, Supplementary Figure S3G, Supplementary Table S2).

The relationship between the different cfDNA quantification methods was evaluated using complementary Spearman correlation and Bland–Altman agreement analyses in the overall cohort and separately in BC patients and HC. Spearman correlation analysis demonstrated significant positive associations between Qubit, TS, and gDNA-corrected cfDNA concentrations in the overall cohort as well as within the BC and HC groups. Qubit and gDNA-corrected cfDNA concentrations exhibited the strongest correlations across the overall cohort and subgroup analyses (Spearman’s r = 0.777 overall, 0.682 in BC patients, and 0.834 in HC; all *p* < 0.0001). Moderate positive correlations were observed between Qubit and TS, and between TS and gDNA-corrected cfDNA concentrations in both the overall cohort and the subgroup analyses (Table 1).

Bland–Altman analysis demonstrated a small positive bias of Qubit relative to TapeStation (TS) in the overall cohort (0.245 ng/µL), BC patients (0.303 ng/µL), and HC (0.162 ng/µL), indicating that fluorometric quantification generally yielded slightly higher cfDNA concentrations than electrophoretic quantification. Following genomic DNA correction, the mean bias between Qubit and corrected cfDNA concentrations decreased across all analyses (0.182 ng/µL overall, 0.240 ng/µL in BC, and 0.098 ng/µL in HC). The comparison between TS and gDNA-corrected cfDNA concentrations showed the smallest mean bias (~0.06 ng/µL) together with the narrowest 95% LoA across the overall cohort and subgroup analyses, indicating the closest agreement among the three cfDNA quantification methods (Table 1). Overall, the relatively small mean biases and narrow 95% LoA indicate reasonable agreement among the cfDNA quantification methods.

### 3.3. Measurement of cfDNA Integrity Is Method Dependent

In addition to the DII (ALU), cfDNA integrity was independently assessed using TS electrophoretic fragmentation profiles by calculating the TS-derived DNA Integrity Index DII (TS), defined as the ratio of di-/tri-nucleosomal fragments (301–1000 bp) (long) to mono-nucleosomal fragments (101–300 bp) (short), representing a long-to-short fragment ratio. Although the median DII (TS) was higher in BC patients than in HC, the difference was not statistically significant (*p* = 0.1828; Figure 2B, Supplementary Table S2). Consistent with this finding, the Hodges–Lehmann median difference was 0.029 (95% CI: −0.0137 to 0.0714), indicating a small estimated between-group difference with the confidence interval including zero. Similarly, concentrations of mono- and di-/tri-nucleosomal cfDNA fragments as well as di-/tri-nucleosomal fragment size did not differ significantly between the two groups. However, mono-nucleosomal fragment size was significantly lower in BC patients than in HC (193 vs. 201 bp, *p* < 0.0001; Table 2).

To compare the two approaches for assessing cfDNA integrity, the relationship between DII (ALU) and DII (TS) was evaluated using Spearman correlation and Bland–Altman analyses. Spearman correlation analysis showed no significant association between DII (ALU) and DII (TS) in the combined cohort (r = −0.112, *p* = 0.148), HC (r = −0.075, *p* = 0.538), or BC patients (r = −0.170, *p* = 0.091) (Table 1, Figure 2C). Bland–Altman analysis likewise demonstrated poor agreement between the two methods across all analyses, as evidenced by relatively large mean biases and wide 95% LoA, with a mean bias of −0.683 (95% LoA: −2.277 to 0.912) in the combined cohort, −0.409 (95% LoA: −1.381 to 0.562) in HC, and −0.871 (95% LoA: −2.696 to 0.953) in BC patients (Table 1).

### 3.4. Evaluation of the Influence of Residual Genomic DNA Contamination on ALU-Based Biomarkers

To further evaluate the potential influence of residual genomic DNA contamination on ALU-based biomarkers, correlation analyses were performed between the estimated gDNA% and ALU247 concentration as well as the DII (ALU). No significant correlation was observed between gDNA% and ALU247 concentration in the combined cohort, whereas a weak positive correlation was observed for DII (ALU). Within the BC cohort, moderate positive correlations were observed between gDNA% and both ALU247 concentration and DII (ALU). In contrast, no consistent positive association was observed in HC (Supplementary Table S4).

To further assess the influence of residual genomic DNA on the observed between-group differences, a sensitivity analysis was performed by excluding the 5% and 10% of samples with the highest estimated gDNA%. Following exclusion of the 5% and 10% of samples with the highest estimated gDNA%, ALU247 concentrations remained significantly higher in BC patients than in HC (5% exclusion: median [Q1–Q3], 0.105 [0.066–0.189] vs. 0.055 [0.039–0.079] ng/µL; 10% exclusion: 0.103 [0.063–0.170] vs. 0.056 [0.041–0.079] ng/µL; both *p* < 0.0001). Similarly, ALU-DII remained significantly elevated following exclusion of the top 5% (0.982 [0.524–1.504] vs. 0.718 [0.362–1.056]; *p* = 0.0015) and top 10% (0.914 [0.523–1.491] vs. 0.738 [0.360–1.104]; *p* = 0.0077) of samples with the highest estimated gDNA%. Although exclusion of samples with the highest estimated gDNA% resulted in a slight attenuation of the Hodges–Lehmann median differences and rank-biserial correlation coefficients overall, ALU247 retained a comparatively stronger effect size than DII (ALU) following both 5% and 10% exclusions (r > 0.5). Importantly, the differences between BC patients and HC remained statistically significant across all analyses (Supplementary Table S5).

In the exploratory covariate-adjusted analysis, estimated gDNA% was significantly associated with both ALU247 (β = 0.00746, *p* < 0.001) and DII (ALU) (β = 0.0135, *p* < 0.001). After adjustment for estimated gDNA%, the Tumor–Control differences remained significant for ALU247 (β = 0.141, *p* = 0.011) and DII (ALU) (β = 0.479, *p* < 0.001) (Supplementary Table S6).

## 4. Discussion

Circulating cell-free DNA (cfDNA) has emerged as a promising analyte for liquid biopsy because its concentration and fragmentation patterns reflect biological processes associated with tumor development, including increased cellular turnover, apoptosis, and necrosis [11,17,18,19]. However, despite growing interest in cfDNA analysis, variability among analytical platforms and the influence of residual gDNA contamination remain important challenges that limit the standardization of cfDNA measurements. In the present exploratory study, we systematically compared three complementary approaches for cfDNA quantification and integrity assessment—Qubit fluorometry, TapeStation electrophoretic profiling, and ALU-based qPCR—within the same breast cancer and healthy control cohort. This direct comparison enabled evaluation of how the three analytical methods characterized cfDNA in BC patients and HC. Although these platforms rely on different analytical principles, they provided complementary information on cfDNA quantity and fragmentation. Within the ALU-based qPCR measurements, ALU247 and DII (ALU) were significantly higher in BC patients, whereas ALU115 did not differ significantly between groups, highlighting important methodological considerations regarding cfDNA quantification and integrity assessment.

Among the methods evaluated, Qubit fluorometry yielded the highest estimates of total DNA concentration. This observation was expected because fluorometric assays quantify all double-stranded DNA present in plasma and do not distinguish circulating cfDNA from residual high-molecular-weight genomic DNA introduced during blood collection or sample processing. Residual genomic DNA contamination is a recognized limitation of fluorometric quantification because even small amounts of leukocyte-derived DNA may lead to overestimation of cfDNA concentration [20]. Electrophoretic platforms such as TapeStation (TS) overcome this limitation by separating DNA fragments according to size, thereby providing both concentration and fragment-size information. In the present study, electrophoretic profiling demonstrated the presence of high-molecular-weight DNA fragments in both BC patients and HC, suggesting residual genomic DNA contamination. We therefore used the proportion of DNA fragments >1 kb obtained from the TS electropherogram as an operational estimate of the high-molecular-weight DNA fraction to derive an exploratory gDNA-corrected cfDNA concentration. Notably, corrected cfDNA concentrations remained significantly higher in BC patients than in HC, indicating that the observed differences were not solely attributable to high-molecular-weight DNA contamination. Across all significant cfDNA quantification approaches, the Hodges–Lehmann median differences and corresponding 95% CI consistently indicated higher cfDNA concentrations in BC patients than in HC, supporting the robustness of the observed between-group differences.

Spearman correlation analyses demonstrated significant positive associations among Qubit, TS, and gDNA-corrected cfDNA concentrations. Bland–Altman analysis further showed that genomic DNA correction reduced the mean bias between fluorometric and electrophoretic measurements, improving their comparability. These findings suggest that a simple electrophoretic-based correction may improve the interpretation of Qubit-derived cfDNA measurements while retaining the speed, simplicity, and widespread availability of fluorometric quantification. However, TS remains valuable because, in addition to quantification, it provides information on cfDNA fragment-size distribution that cannot be obtained by Qubit alone. The proposed correction strategy should be interpreted cautiously. Because the correction factor was derived from the TS electropherogram itself, the improved agreement should be interpreted as an exploratory methodological observation rather than independent validation of the correction strategy. Moreover, because the correction was based solely on DNA fragments >1 kb, it provides only an approximate estimate of residual genomic DNA contamination, as smaller sheared genomic DNA fragments are not captured by this approach. Future studies incorporating orthogonal approaches, such as single-copy-gene qPCR/ddPCR or sequencing-based fragmentomic analyses, will be important to further evaluate and refine this correction strategy.

Exploratory subgroup analyses demonstrated no statistically significant differences in cfDNA concentrations across age groups, tumor stage, or molecular subtypes after adjustment for multiple comparisons, although numerical differences were observed among several clinicopathological subgroups. Consistent with these findings, effect size estimates were small across all subgroup analyses. However, these exploratory subgroup findings should be interpreted cautiously because the study was not powered to detect subgroup-specific differences, particularly within the HER2+ and TNBC cohorts, which comprised relatively small numbers of patients.

In addition to total cfDNA quantification, we evaluated ALU-based qPCR as a complementary approach for assessing cfDNA quantification and fragmentation. ALU repetitive elements are highly abundant throughout the human genome and therefore provide a sensitive means of quantifying circulating DNA. The shorter ALU115 amplicon represents the total amplifiable cfDNA pool, whereas the longer ALU247 amplicon preferentially amplifies longer DNA fragments, which are thought to arise from increased necrosis and other non-apoptotic mechanisms of cell death associated with tumor progression [7,21,22,23]. Consequently, the DII (ALU), calculated as the ALU247/ALU115 ratio, provides an indirect measure of altered cfDNA fragmentation. Though ALU115 concentration was not significantly different between groups, both ALU247 concentration and DII (ALU) were significantly higher in BC patients than in HC. The corresponding Hodges–Lehmann median differences and 95% confidence intervals further quantified the magnitude of the increases in ALU247 concentration and ALU-derived DNA integrity in BC patients compared with HC. These findings are consistent with our previous findings and earlier reports supporting the presence of altered cfDNA fragmentation associated with breast cancer [7,15].

Recent sequencing-based fragmentomic studies have demonstrated that tumor-derived cfDNA is preferentially enriched among shorter DNA fragments (<150 bp), whereas the predominant circulating cfDNA population in both healthy individuals and cancer patients consists of mononucleosomal fragments of approximately 166 bp [24,25]. In our TapeStation analysis, a significantly shorter median fragment size was observed in BC patients than in HC (193 vs. 201 bp, *p* < 0.0001). This shift toward shorter mono-nucleosomal fragments may reflect altered cfDNA fragmentation or nucleosomal processing associated with cancer, while the underlying mechanism cannot be determined from the present electrophoretic analysis. Although this observation may initially appear inconsistent with the ALU-based integrity findings, the two approaches interrogate different characteristics of cfDNA fragmentation. Sequencing provides a genome-wide assessment of fragment-length distributions, whereas ALU-based qPCR quantifies predefined repetitive genomic targets of different amplicon lengths (ALU115 and ALU247) to estimate DNA integrity. Thus, ALU-derived DNA integrity reflects the relative abundance of longer versus shorter amplifiable cfDNA fragments rather than the genome-wide fragment-size profile or the abundance of tumor-derived cfDNA. Accordingly, ALU-derived DNA integrity should be interpreted as an indirect measure of altered cfDNA fragmentation rather than a direct measure of tumor-derived cfDNA abundance.

To determine whether a comparable integrity index could be obtained from electrophoretic fragment-size profiling, we evaluated the DII (TS). Although DII (TS) showed numerically higher values in BC patients than in HC, the difference was not statistically significant. Furthermore, no significant association was observed between DII (ALU) and DII (TS), and Bland–Altman analysis demonstrated poor agreement between the two methods, with wide LoA. These findings are not unexpected because the two approaches are based on different analytical principles and fragment definitions. ALU-derived DII is calculated as the ALU247/ALU115 ratio, where ALU115 represents the total amplifiable cfDNA pool (≥115 bp) and ALU247 amplifies fragments ≥247 bp [7,20,21,22]. In contrast, DII (TS) is derived from electrophoretic fragment-size distributions and represents the ratio of long (301–1000 bp) to short (101–300 bp) cfDNA fragments. Importantly, fragments between 247 and 301 bp contribute to the ALU247 signal but are classified within the short-fragment interval used to calculate DII (TS). Consequently, the two approaches quantify overlapping, but not identical, cfDNA fragment populations. The poor agreement observed by Bland–Altman analysis further supports the conclusion that these methods should be regarded as complementary rather than directly interchangeable measures of cfDNA integrity.

Unlike Qubit-derived cfDNA concentrations, direct correction of ALU-based measurements for residual genomic DNA was not feasible. We therefore evaluated the potential influence of residual gDNA by correlating the estimated gDNA% with ALU247 concentration and DII (ALU) and by performing sensitivity analyses after excluding samples with the highest estimated gDNA%. Moderate positive correlations observed within the BC cohort suggest that residual genomic DNA contributes to variability in ALU-based measurements. Interestingly, estimated gDNA% showed a weak negative correlation with ALU247 in the HC group (r = −0.328, *p* = 0.006), which was not consistent with a simple contamination-driven increase in ALU247 and may reflect assay behavior at lower template concentrations. However, both ALU247 concentration and DII (ALU) remained significantly higher in BC patients than in HC following exclusion of the most highly contaminated samples. Although the Hodges–Lehmann median differences and rank-biserial correlation coefficients were modestly attenuated overall, ALU247 consistently retained larger effect sizes than DII (ALU) following both 5% and 10% exclusions. Similarly, although estimated gDNA% remained significantly associated with both measurements, the BC–HC differences remained significant for both ALU247 and DII (ALU) after adjustment for estimated gDNA%. Collectively, these findings suggest that residual genomic DNA contributes to variability in ALU-based measurements but does not fully account for the observed differences between BC patients and HC.

This study has several limitations. First, orthogonal molecular validation was limited. Although targeted NGS was performed in a small subset of samples, tumor-associated variants were detected in only one case. Consequently, ctDNA could not be comprehensively confirmed across the study cohort, and the observed alterations in cfDNA concentration and fragmentation should be interpreted as tumor-associated cfDNA features rather than direct evidence of circulating tumor DNA. Second, the study did not include sequencing-based fragmentomic analyses or orthogonal validation using highly sensitive methods such as droplet digital PCR (ddPCR), which may provide additional insights into cfDNA fragmentation patterns and tumor-derived DNA. Third, the healthy control cohort was not age- or sex-matched to the breast cancer cohort, which may have introduced potential confounding. To assess the potential influence of sex imbalance, a female-only sensitivity analysis was performed. The significant differences observed in the primary analysis were retained, with comparable or greater effect sizes than those observed in the combined analysis (Supplementary Table S7), suggesting that the observed findings were not substantially driven by the sex imbalance in the control cohort. Nevertheless, future studies incorporating larger, age- and sex-matched cohorts, benign breast disease controls, and comprehensive orthogonal molecular validation, including sequencing-based fragmentomics and droplet digital PCR (ddPCR), will be important to further validate and standardize cfDNA quantification and integrity assessment across analytical platforms.

## 5. Conclusions

This exploratory study demonstrates that Qubit fluorometry, TapeStation (TS) electrophoretic profiling, and ALU-based qPCR provide complementary information on circulating cfDNA and consistently discriminate between BC patients and HC. While fluorometry provides rapid cfDNA quantification, TS enables assessment of fragment-size distribution and genomic DNA contamination, and ALU-based qPCR, particularly ALU247 and DII (ALU), provides complementary information on cfDNA fragmentation and integrity. The findings further demonstrate that cfDNA integrity measurements are method dependent and should be interpreted within the context of the analytical platform rather than considered directly interchangeable. Collectively, these results provide a methodological framework for future studies aimed at standardizing cfDNA quantification and integrity assessment for liquid biopsy applications.

## Figures and Tables

**Table 1:** Spearman correlation and Bland–Altman agreement analyses of cfDNA quantification and integrity assessment methods. Mean bias was calculated as the difference between the first and second methods listed in each comparison (first method − second method). Positive values indicate that the first method yielded higher measurements on average, whereas negative values indicate that the second method yielded higher measurements. The magnitude of the mean bias (i.e., its closeness to zero), rather than its sign, reflects the degree of systematic difference between methods, with values closer to zero indicating better agreement. Narrower 95% limits of agreement (LoA) indicate better agreement between methods.

Comparison	Group	Spearman’s r	*p* Value	Bland–Altman Mean Bias	95% Limits of Agreement
Qubit vs. TS	All	0.546	<0.0001	0.245	−0.338 to 0.829
	BC patients	0.609	<0.0001	0.303	−0.417 to 1.023
	HC	0.323	0.0068	0.162	−0.043 to 0.367
Qubit vs. gDNA-corrected cfDNA	All	0.777	<0.0001	0.182	−0.498 to 0.862
	BC patients	0.682	<0.0001	0.240	−0.621 to 1.101
	HC	0.834	<0.0001	0.098	−0.030 to 0.226
TS vs. gDNA-corrected cfDNA	All	0.598	<0.0001	−0.063	−0.372 to 0.246
	BC patients	0.534	<0.0001	−0.063	−0.447 to 0.321
	HC	0.549	<0.0001	−0.064	−0.210 to 0.083
DII (TS) vs. DII (ALU)	All	−0.112	0.148	−0.683	−2.277 to 0.912
	BC patients	−0.170	0.091	−0.871	−2.696 to 0.953
	HC	−0.075	0.539	−0.409	−1.381 to 0.562

BC: Breast Cancer, HC: Healthy Control, TS: TapeStation; gDNA: genomic DNA; DII: DNA Integrity Index.

**Table 2:** Comparison of TapeStation-derived cfDNA fragment size characteristics between BC patients and HC. Data are presented as median (Q1–Q3). Comparisons between groups were performed using the Mann–Whitney U test. Hodges–Lehmann median differences were calculated as BC − HC and are reported with corresponding 95% confidence intervals (CI). Positive values indicate higher median values in BC patients than in HC, whereas negative values indicate lower median values in BC patients than in HC. A 95% CI that does not include 0 indicates a statistically significant difference between the groups.

Parameter	BC Patients (Median [Q1–Q3])	HC (Median [Q1–Q3])	*p* Value	Hodges-Lehmann Median Difference	95% CI
Mono-nucleosomal concentration (ng/µL)	0.094 (0.063–0.150)	0.087 (0.056–0.123)	0.212	0.011	−0.0060 to 0.0280
Di-/tri-nucleosomal concentration (ng/µL)	0.033 (0.018–0.056)	0.029 (0.012–0.046)	0.067	0.005	−0.0004 to 0.0132
Mono-nucleosomal fragment size (bp)	193 (189–200)	201 (199–204)	<0.0001	−8.000	−10.000 to −6.0001
Di-/tri-nucleosomal fragment size (bp)	554 (533–573)	542 (527–561)	0.072	9.000	−1.000 to 19.000

BC: Breast Cancer, HC: Healthy Control.

## Data Availability

The data presented in this study are not publicly available due to ethical and privacy restrictions concerning participant-level information but may be available from the corresponding author upon reasonable request and subject to appropriate institutional and ethical approvals.

## Supplementary Materials

The following supporting information can be downloaded at: https://www.mdpi.com/article/10.3390/mps9050124/s1. Supplementary Methods; Table S1. Clinico-pathological characteristics of cohort; Table S2. Comparison of cfDNA concentration and DNA integrity parameters between BC patients and HC; Table S3. Subgroup analysis of plasma cfDNA concentration measured by Qubit according to age category, tumour stage, and molecular subtype in BC patients; Table S4. Spearman correlation analysis between the estimated genomic DNA fraction (gDNA%) and ALU-based biomarkers; Table S5. Sensitivity analysis evaluating the effect of excluding the 5% and 10% of samples with the highest estimated genomic DNA fraction (gDNA%); Table S6. Exploratory linear regression analysis of ALU-based measurements with estimated gDNA% as a covariate; Table S7. Sensitivity analysis according to sex composition of the control group; Figure S1. Stratified analysis of plasma cfDNA concentrations in BC patients; Figure S2. Comparison of ALU115 concentration between HC and BC patients; Figure S3. TS fragment profiles and gDNA-corrected cfDNA concentration.
