# Supplementary material for: Evaluating Cell-Free DNA Quantification and Integrity in Breast Cancer Using Fluorometric, Electrophoretic and PCR-Based Platforms
Source: Methods Protoc. 2026 Aug 24;9(5):124. doi: 10.3390/mps9050124 (PMC13600121; doi:10.3390/mps9050124)
Supplement: Supplementary file 1 [file mps-09-00124-s001.zip › mps-4451659-supplementary.pdf]

methods and protocols

MDPI

Evaluating cell-free DNA quantification and integrity in breast cancer using fluorometric, electrophoretic and PCR-based platforms

Supplementary Methods

qPCR assay conditions: Quantitative real-time PCR was performed using SYBR Green chemistry. Thermal cycling consisted of an initial denaturation at 95°C for 10 min, followed by 40 cycles of denaturation at 95°C for 15 s, annealing at 60°C for 15 s, and extension at 72°C for 15 s. A melt curve analysis was performed after amplification to verify product specificity.

Absolute quantification was performed using serial dilutions of human genomic DNA (10 ng to 0.01 pg) following the previously validated protocol. Assay optimization and standard curve validation were performed in the original study; therefore, these parameters were not re-evaluated in the present study.

Quality control: All samples were analyzed in duplicate. Amplification specificity was confirmed by melt-curve analysis, yielding a single peak at approximately 76°C for ALU115 and 87°C for ALU247.

Supplementary Table S1. Clinico-pathological characteristics of cohort

Characteristics		BC patients $n = 100$	HC $n = 70$
**Age**	≤50	27	61
	>50	73	9
**Gender**	Male	-	36
	Female	100	34
**Subtype**	HR+	62	-
	HER2+	22	-
	TNBC	16	-
**Tumor Size**	≤2 cm	38	-
	>2 cm	62	-
**LN Status**	Positive	38	-
	Negative	62	-
**Grade**	1	13	-
	2	53	-
	3	34	-

**Clinical Stage**	I	22	-
	II	52	-
	III	26	-

BC: Breast Cancer, HC: Healthy Control; HR+: Hormone Receptor Positive, TNBC: Triple Negative Breast Cancer; LN status: Lymph Node status

Supplementary Table S2. Comparison of cfDNA concentration and DNA integrity parameters between BC patients and HC. Data are presented as median (Q1–Q3). Comparisons between groups were performed using the Mann–Whitney U test. Effect sizes are reported as Hodges–Lehmann median differences (BC - HC) with corresponding 95% confidence intervals (CI). A 95% CI that does not include 0 supports a difference between the groups. gDNA: genomic DNA; DII: DNA Integrity Index.

**cfDNA concentration**	**BC patients (Median [Q1–Q3]) (ng/µL)**	**HC (Median [Q1–Q3]) (ng/µL)**	***p* value**	**Hodges-Lehmann median difference**	**95% CI**
Qubit	0.329 (0.245–0.490)	0.240 (0.174–0.300)	<0.0001	0.098	0.0570 to 0.1441
TapeStation	0.109 (0.065–0.170)	0.084 (0.053–0.110)	0.0048	0.029	0.0094 to 0.0500
gDNA corrected cfDNA	0.195 (0.132–0.284)	0.150 (0.093–0.191)	<0.0001	0.056	0.0287 to 0.0871
ALU247	0.113 (0.070–0.228)	0.055 (0.039–0.079)	<0.0001	0.058	0.0389 to 0.0808
DII (ALU)	1.074 (0.531–1.522)	0.714 (0.364–1.040)	0.0002	0.338	0.1651 to 0.5310
DII (TS)	0.343 (0.259–0.448)	0.313 (0.247–0.371)	0.1828	0.029	-0.0137 to 0.0714

gDNA: genomic DNA; DII: DNA Integrity Index

Supplementary Table S3. Subgroup analysis of plasma cfDNA concentration measured by Qubit according to age category, tumour stage, and molecular subtype in BC patients. Data are presented as median (Q1–Q3). Comparisons between two groups (age and stage) were performed using the Mann–Whitney U test, whereas molecular subtype was analysed using the Kruskal–Wallis test. p values were adjusted for multiple subgroup comparisons using the Benjamini–Hochberg (BH) procedure. Effect sizes are reported as the rank-biserial correlation (*r*) for Mann–Whitney U tests and epsilon squared (ε^2) for the Kruskal–Wallis test.

**Comparison**	**Group**	**Median (Q1–Q3)**	***p* value**	**BH-adjusted *p* value**	**Effect size (r/ε^2)**
Age	≤50	0.263 (0.233–0.422)	0.059	0.100	r = 0.247

	>50	0.351 (0.250–0.498)			
Stage	Early	0.307 (0.240–0.479)	0.093	0.100	r = 0.222
	Late	0.400 (0.279–0.621)			
Subtype	HR+	0.351 (0.247–0.501)	0.100	0.100	$\varepsilon^2 = 0.027$
	HER2+	0.330 (0.252–0.450)			
	TNBC	0.256 (0.201–0.351)			

HR+: Hormone Receptor Positive, TNBC: Triple Negative Breast Cancer

Supplementary Table S4. Spearman correlation analysis between the estimated genomic DNA fraction (gDNA%) and ALU-based biomarkers. Correlations were evaluated for the combined cohort (all) as well as for BC patients, and HC separately.

Biomarker	Dataset	Spearman's *r*	*p* value
ALU247	All samples (*n* = 170)	0.106	0.169
	BC patients (*n* = 100)	0.411	<0.0001
	HC (*n* = 70)	-0.328	0.0060
DII (ALU)	All samples (*n* = 170)	0.280	0.0002
	BC patients (*n* = 100)	0.443	<0.0001
	HC (*n* = 70)	0.032	0.791

BC: Breast Cancer; HC: Healthy controls; DII: DNA Integrity Index

Supplementary Table S5. Sensitivity analysis evaluating the effect of excluding the 5% and 10% of samples with the highest estimated genomic DNA fraction (gDNA%). Analyses were performed for the combined cohort (all), BC patients, and HC separately. For each exclusion scenario, Mann–Whitney U tests were used to compare biomarker values between BC patients and HC. Data are presented as median (Q1–Q3). Effect sizes are reported as Hodges–Lehmann median differences (BC − HC) with corresponding 95% confidence intervals (CI) and rank-biserial correlation coefficients. Positive Hodges–Lehmann values indicate higher median values in BC patients than in HC. A 95% CI that does not include 0 supports a difference between the groups. Rank-biserial correlation coefficients range from -1 to +1, with values farther from 0 indicating larger effect sizes.

Biomarker	Dataset	BC patients Median (Q1–Q3)	HC Median (Q1–Q3)	*p* value	Hodges–Lehmann median difference	95% CI	Rank-biserial correlation coefficient (r)
ALU247	All samples (*n* = 170)	0.113 (0.070–0.228)	0.055 (0.039–0.079)	<0.0001	0.058	0.039 to 0.081	0.572

	Top 5% gDNA removed (n = 162)	0.105 (0.066–0.189)	0.055 (0.039–0.079)	<0.0001	0.048	0.032 to 0.068	0.531
	Top 10% gDNA removed (n = 153)	0.103 (0.063–0.170)	0.056 (0.041–0.079)	<0.0001	0.044	0.028 to 0.062	0.507
DII (ALU)	All samples (n = 170)	1.075 (0.531–1.522)	0.714 (0.364–1.040)	0.0002	0.338	0.165 to 0.531	0.337
	Top 5% gDNA removed (n = 162)	0.982 (0.524–1.504)	0.718 (0.362–1.056)	0.0015	0.285	0.114 to 0.475	0.294
	Top 10% gDNA removed (n = 153)	0.914 (0.523–1.491)	0.738 (0.360–1.104)	0.0077	0.249	0.068 to 0.423	0.254

BC: Breast Cancer; HC: Healthy Control; DII: DNA Integrity Index

Supplementary Table S6. Exploratory linear regression analysis of ALU-based measurements with estimated gDNA% as a covariate. β represents the regression coefficient from a linear model including sample group (Tumor vs Control) and estimated gDNA% as covariates. For the Tumor vs Control comparison, a positive β indicates higher ALU247 or DII (ALU) in tumors after adjustment for estimated gDNA%.

Outcome	Predictor	Adjusted estimate (β)	p value
ALU247	Tumor vs Control	0.141	0.011
	Estimated gDNA%	0.00746	<0.001
DII (ALU)	Tumor vs Control	0.479	<0.001
	Estimated gDNA%	0.0135	<0.001

Supplementary Table S7. Sensitivity analysis according to sex composition of the control group. Data are presented as median (Q1–Q3). Comparisons were performed using the Mann–Whitney U test. Rank-biserial correlation (r) was used as a measure of effect size. "All participants" includes male and female participants combined in HC, whereas "Female-only" includes female participants only in HC.

Biomarker	Analysis	BC median (Q1–Q3)	HC median (Q1–Q3)	p value	Rank-biserial correlation

					**coefficient (r)**
TS	All participants	0.109 (0.065–0.170)	0.084 (0.053–0.110)	0.0048	0.256
	Female-only		0.073 (0.044–0.099)	0.0013	0.371
Qubit	All participants	0.329 (0.245–0.490)	0.240 (0.174–0.300)	<0.0001	0.439
	Female-only		0.242 (0.179–0.291)	0.0001	0.444
DII (ALU)	All participants	1.074 (0.531–1.522)	0.714 (0.364–1.040)	0.0002	0.337
	Female-only		0.734 (0.358–1.105)	0.0023	0.350
ALU247	All participants	0.113 (0.070–0.228)	0.055 (0.039–0.079)	<0.0001	0.572
	Female-only		0.050 (0.037–0.062)	<0.0001	0.634

BC: Breast Cancer; HC: Healthy Control

Supplementary Figures

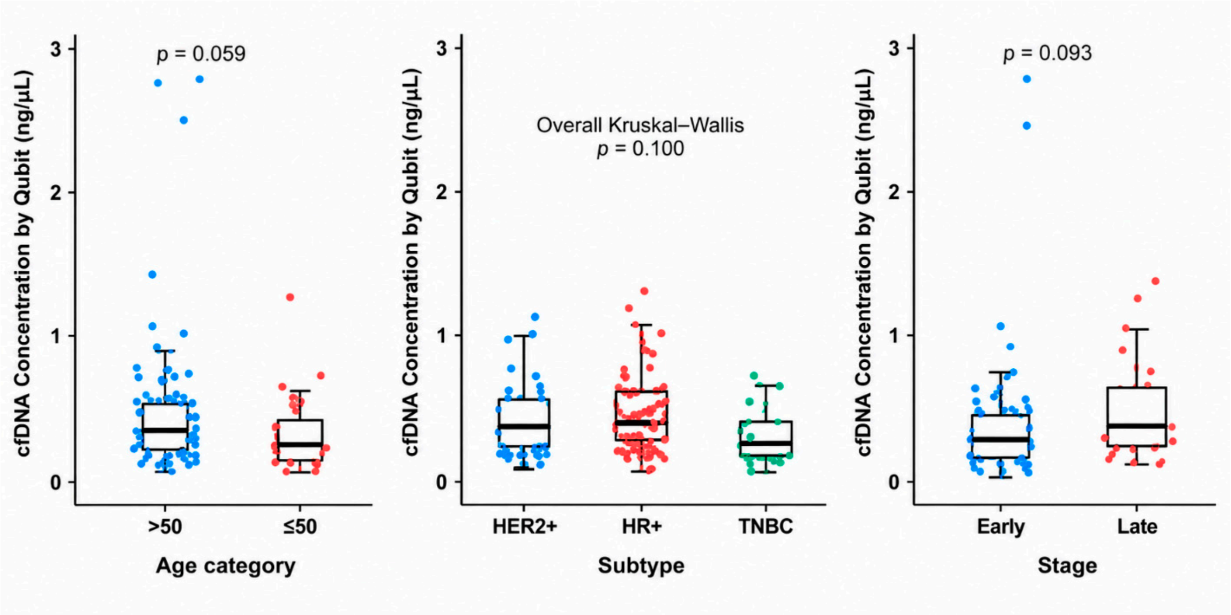

Supplementary Figure S1. Stratified analysis of plasma cfDNA concentrations in BC patients. (A) Comparison of plasma cfDNA concentrations between patients aged ≤50 years and >50 years. Although median cfDNA concentrations were numerically higher in patients aged >50 years, the difference was not statistically significant (Mann–Whitney U test, $p = 0.059$). (B) Comparison of plasma cfDNA concentrations across molecular subtypes (HR+, HER2+, and TNBC). No significant differences were observed among the molecular subtypes (overall Kruskal–Wallis test, $p = 0.100$). (C) Comparison of plasma cfDNA concentrations between early- and late-stage BC patients. Although median cfDNA concentrations were numerically higher in patients with late-stage disease, the difference was not statistically significant (Mann–Whitney U test, $p = 0.093$). Following Benjamini–Hochberg (BH) adjustment for the prespecified subgroup

analyses, none of the comparisons remained statistically significant (BH-adjusted $p = 0.100$ for all comparisons). Individual observations are shown as dots; the box represents the interquartile range (IQR; Q1–Q3), the horizontal line indicates the median.

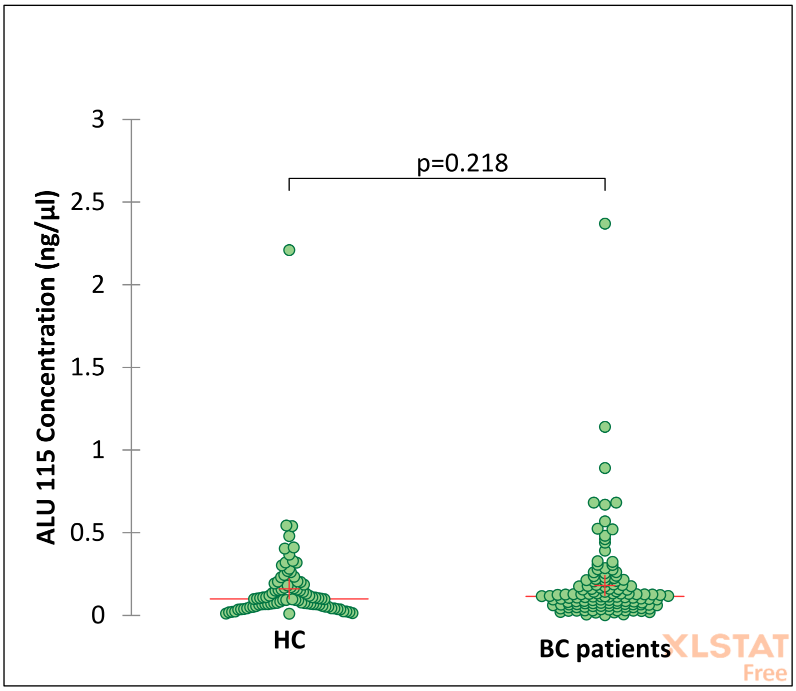

Supplementary Figure S2. Comparison of ALU115 concentration between HC and BC patients. Differences between groups were assessed using the Mann–Whitney U test. No statistically significant difference was observed ($p = 0.218$). HC: Healthy Control, BC: Breast Cancer

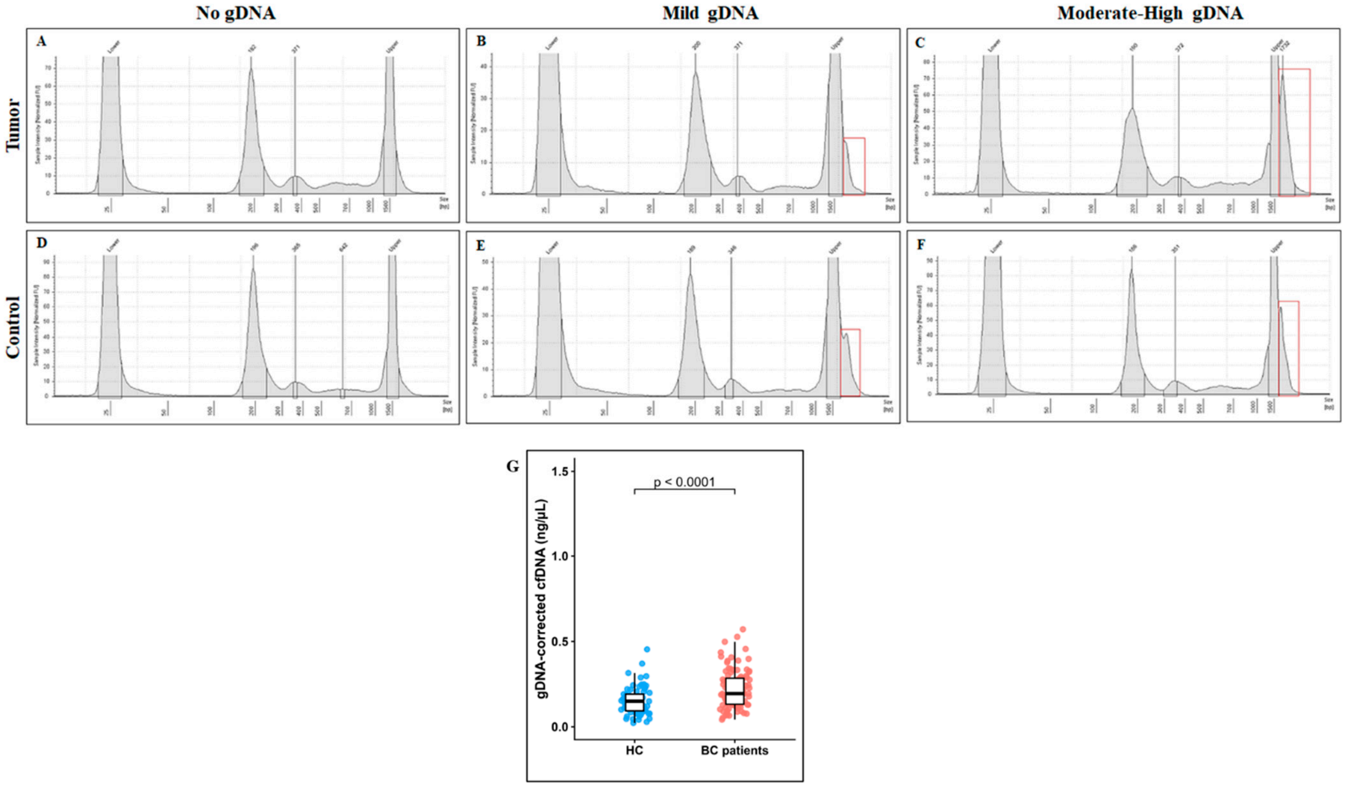

Supplementary Figure S3. TS fragment profiles and gDNA-corrected cfDNA concentration. Representative electropherograms illustrate the range of cfDNA fragment profiles and varying degrees of residual genomic DNA (gDNA) contamination observed in the BC patient (Tumor) and HC (Control) groups. (A-C) Representative TS electropherograms from Tumor samples showing cfDNA fragment distributions with prominent mono- and di-nucleosomal peaks and varying degrees of gDNA contamination: no detectable gDNA (A), mild gDNA contamination (B), and moderate-to-high gDNA contamination (C). (D-F) Corresponding electropherograms from Control samples showing no detectable gDNA (D), mild gDNA contamination (E), and moderate-to-high gDNA contamination (F). High-molecular-weight gDNA fragments are highlighted by red boxes. (G) gDNA-corrected cfDNA concentrations are significantly higher in BC patients compared to HC ($p < 0.0001$). For boxplot G, individual observations are shown as dots; the box represents the interquartile range (IQR; Q1–Q3), the horizontal line indicates the median.

HC: Healthy Control, BC: Breast Cancer.
